# Evaluation of implementation and effectiveness of neck-specific exercise for persistent disability and pain after whiplash injury: study protocol for a randomized controlled study using a hybrid 2 design

**DOI:** 10.1186/s12891-022-05470-y

**Published:** 2022-05-30

**Authors:** Gunnel Peterson, Siw Carlfjord, Emma Nilsing Strid, Sofia Ask, Margaretha Jönsson, Anneli Peolsson

**Affiliations:** 1 Center for Clinical Research Sörmland, Uppsala University, Eskilstuna, SE Sweden; 2 Department of Health, Medicine and Caring Sciences, Unit of Physiotherapy, Linköping University, Linköping, Sweden; 3 Department of Health, Medicine and Caring Sciences, Division of Society and Health, Linköping University, Linköping, Sweden; 4 University Health Care Research Center, Faculty of Medicine and Health, Örebro University, Örebro, Sweden; 5 Prima Health care Center, Västerås, County Council Västmanland Sweden; 6 Occupational and Environmental Medicine Center, Department of Health, Medicine and Caring Sciences, Unit of Clinical Medicine, Linköping University, Linköping, Sweden

**Keywords:** Neck pain, Exercise, Whiplash injuries, Implementation science, Rehabilitation

## Abstract

**Background:**

Persistent pain and disability in whiplash-associated disorders (WAD) grades II and III are common. In two randomized controlled trials (RCTs) of neck-specific exercises (NSE), we have seen promising results in chronic WAD, with a sustained clinically important reduction in pain and disability. NSE can also be delivered through internet support (NSEIT) and a few visits to a physiotherapist, saving time and cost for both patients and providers. NSE have been shown to have positive effects in other neck pain disorders and we will evaluate the diffusion of the exercises to other patients. The aims of the proposed study are to evaluate an implementation strategy for NSEIT and NSE in primary health care and to evaluate the effectiveness of NSEIT and NSE in clinical practice.

**Methods:**

The proposed study is a prospective cluster-randomized mixed-design study with hybrid 2 trial design. Reg. physiotherapists working in twenty physiotherapy clinics will be included. The primary implementation outcome is proportion of patients with neck pain receiving neck-specific exercise. Secondary outcomes are; physiotherapists attitudes to implementation of evidence-based practice, their self-efficacy and confidence in performing NSEIT/NSE, number of patients visits, and use of additional or other exercises or treatment. To further evaluate the implementation strategy, two qualitative studies will be performed with a sample of the physiotherapists. The primary outcome in the patient effectiveness evaluation is self-reported neck disability according to the Neck Disability Index (NDI). Secondary outcomes are pain intensity in the neck, arm, and head; dizziness; work- and health-related issues; and patient’s improvement or deterioration over time. All measurements will be conducted at baseline and at 3 and 12 months. Physiotherapists´ self-efficacy and confidence in diagnosing and treating patients with neck pain will also be evaluated directly after their instruction in NSEIT/NSE.

**Discussion:**

This trial will evaluate the implementation strategy in terms of adoption of and adherence to NSEIT and NSE in clinical primary health care, and measure diffusion of the method to other patients. In parallel, the effectiveness of the method will be evaluated. The results may guide physiotherapists and health care providers to sustainable and effective implementation of effective exercise programs.

**Trial registration:**

The randomized trial is registered on ClinicalTrials.gov, NCT05198258, initial release date January 20, 2022.

## Background

There is usually a considerable time lag from results in health research to implementation and use in clinical practice. Time to implementation is commonly reported to be up to 20 years [1]. For patients suffering from disorders, such as persistent whiplash-associated disorders (WAD), for which treatments until recently have been inconclusive, fast implementation of new effective methods is crucial. Years after whiplash trauma, persistent pain and disability affect up to 50% of those injured [2], and 30% will experience severe symptoms [3]. No evidence is currently available regarding invasive interventions [4]; exercise and patient education are recommended [5], but only modest effects have been demonstrated [6, 7]. Results from a randomized controlled study (RCT) of neck-specific exercises (NSE) showed promising results in chronic WAD, with a sustained clinically important reduction in pain and disability [8]. NSE supervised by a physiotherapist twice weekly for 3 months was superior to prescribed physical activity [9, 10] and better than staying on a waiting list for individuals with chronic WAD [11]. The NSE group showed up to 50% pain reduction and improvement in disability [10], with sustained improvement 2 years after the exercise intervention [8]. In a recent RCT, NSE was compared with an internet-based neck-specific exercise program (NSEIT) that consists of the same information and exercises as the NSE [12] but includes only four visits to the physiotherapist. Although participants had chronic WAD, both groups significantly improved (unpublished results, manuscript under review), and the internet-based intervention was non-inferior to NSE. The results thereby confirm the good results of the earlier RCT [8]. A broad implementation of this method in primary care would give patients with WAD a flexible method with which to improve function and health and decrease pain. NSEIT is also less time consuming and less expensive for the health care system.

### Implementation strategies

Several theories, frameworks, and taxonomies to promote and facilitate the uptake of evidence in practice are currently available [13, 14]. Nonetheless, theory-based efforts to implement new methods into practice have had meager results, often with no or small differences compared with controls [15, 16]. This highlights the complexity of changing health care providers’ and patients’ behavior and their attitudes towards the use of new methods. A clearly defined strategy involving different steps in the complex implementation process could overcome implementation resistance and facilitate knowledge translation [17, 18]. To succeed in implementing new evidence-based methods, it is important to understand the factors that hinder versus promote uptake of the new method [19]. This includes the individuals’ environment: for example, institutional factors (leaders) and public policy [20]. The diffusion-of-innovation theory [21] describes a behavioral change model for understanding and promoting the uptake of “an idea, practice or object that is perceived as new by an individual” and the process “by which an innovation is communicated through certain channels over time among the members of a social system”. Building on this theory, key determinants of diffusion speed and extent have been identified by Glanz et al. [22]. To succeed, the new method should be better than the current one, fit the intended audience, be easy to use, provide observable and easily measurable results, be adoptable with minimal investment in time, and have minimal risk and uncertainty [22]. When a comprehensive facilitation method was used to implement a behavioral medicine approach in primary care, the physiotherapists’ efficacy in using the method increased, but the initial change in clinical action was not maintained at the 3-, 6-, or 12-month follow-up [16]. The conclusions were that intrinsic motivation and continued support are important factors in maintaining behavioral changes [16]. Lack of time and heavy workloads in primary care may also make it difficult to implement complex methods.

In a prior study, NSE was implemented in primary care [23]. A one-day instructional program containing interactive components, which was earlier shown to give better results compared with only didactic education [24], was provided. After the instruction, physiotherapists (87%) reported that they used neck-specific exercises in WAD with significantly higher confidence than before, and these results were sustained at the one-year follow-up [23]. Suggestions for further improving the implementation were to arrange a follow-up session for repetition, or to divide the instruction between two occasions. A limitation of the study was that data were collected only at the group level, so it was not possible to evaluate change on an individual basis. The effectiveness of NSE in clinical practice, including the physiotherapists’ fidelity to the exercise program, was not evaluated and needs to be investigated further.

In implementing interventions, there are several factors that must be considered. One factor is how the characteristics of the intervention are perceived by the potential adopters, and if they see a relative advantage in comparison with current practice [22]. Individuals with persistent WAD are difficult to rehabilitate, they are high consumers of health care services [25], and effective treatment is lacking, which may enhance physiotherapists’ willingness to use the method [6, 7, 26, 27]. There is growing evidence that NSE has a significant effect in chronic whiplash-associated disorders (WAD), including in patients with severe symptoms (neurological signs) after the injury [8, 28]. The internet-based version NSEIT could further facilitate its applicability in the busy and complex setting of primary health care. Moreover, the NSEIT and NSE program is not considered a complex intervention and may therefore be easy to implement in clinical practice.

Education is an important component of implementation strategies, but a comprehensive strategy including many meetings with a facilitator has been found to be too time consuming [29]. In the present study the educational sessions will be limited in time to fit the busy physiotherapists’ workloads and will include mixed interactive and didactic components to enhance their effectiveness [24].

### RE-AIM framework

The RE-AIM framework was developed to identify and evaluate a broad aspect of important factors to translate research results into clinical practice [30, 31]. The five RE-AIM dimensions are Reach (R), Effectiveness (E), Adoption (A), Implementation (I), and Maintenance (M). The five dimensions relate both to the individual (R, E, and M, in the present study to patients with neck pain) and to staff and settings (A, I, and M). The RE-AIM framework [30, 31] will be used to evaluate the implementation strategy in the present study.

### Research needs

In randomized controlled trials, the intervention follows a strict protocol directed to a specific diagnosis and excludes other treatments or exercise intervention during the study period. This is important in evaluating its effectiveness—that is, whether the method is delivered as intended and has the same effect as in the RCTs [8, 12]. NSEIT and NSE have been evaluated in patients with chronic WAD [8, 12], but in clinical practice physiotherapists may use the method in patients with other neck pain disorders. To our knowledge this has not been investigated before, either for chronic WAD as a neck-specific exercise program or as an Internet-based exercise program. There are gaps in our knowledge regarding the best way to implement new exercise methods in primary care and whether or not the method will be used as intended and/or if the implemented method, in this case neck-specific exercises, will be used in patients with other neck pain disorders. Therefore, the proposed study will evaluate both the implementation strategy and the effectiveness of the implemented method in clinical practice [32].

### Aims and research question

The aims of the proposed study are to evaluate an implementation strategy for NSEIT and NSE in primary health care and to evaluate the effectiveness of the programs in clinical practice.

These research questions will be investigated according to the RE-AIM framework:

1. Reach: To what extent do patients with WAD receive NSEIT/NSE in primary care?
2. Effectiveness: Is the NSEIT/NSE program effective at decreasing disability and pain in patients with WAD or non-specific neck pain in primary care? Will the results be the same as in the RCTs at 3 and 12 months of follow-up?
3. Adoption: To what extent do the physiotherapists apply the NSEIT/NSE for patients with WAD or non-specific neck pain?
4. Implementation: To what extent is the intervention delivered according to the NSE protocol (fidelity)?
5. Maintenance: To what extent will physiotherapists continue to use NSEIT/NSE at 12 months of follow-up?

## Method

### Design

We propose a prospective cluster-randomized mixed-design study evaluating the implementation strategy and effectiveness of the NSEIT/NSE. The study is based on hybrid 2 trial design, as described by Curran et al. [33], where both implementation and clinical effectiveness are tested simultaneously. This study has been approved by the Swedish Ethical Review Authority (Dnr: 2021-03383, 2021-06016-02, 2022-00479-02) and will be conducted in accordance with the Declaration of Helsinki.

### Participants and settings

**Fig. 1:**
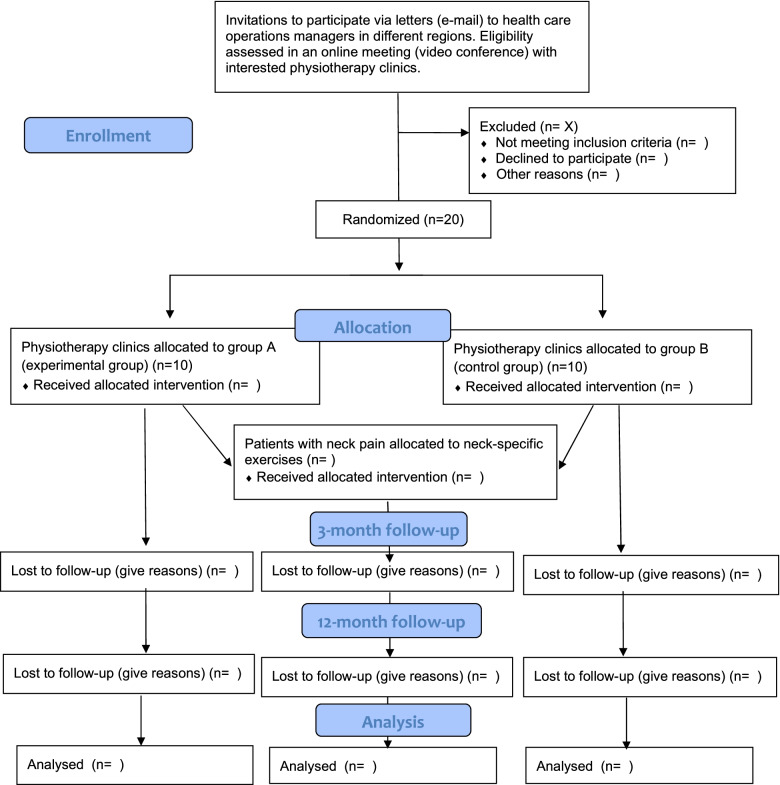
Consort flow diagram

**Fig. 2:**
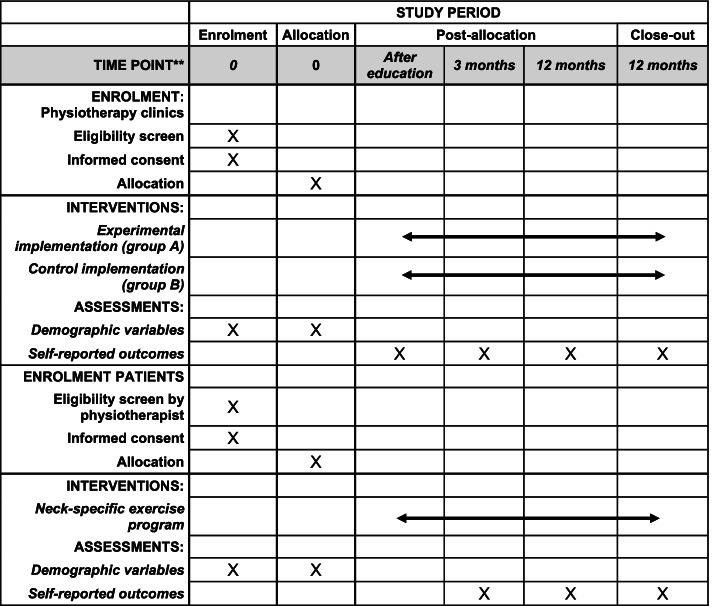
Planned schedule of enrollment, interventions, and assessments

Twenty physiotherapy clinics in primary health care in Sweden will be included. Twenty-five patients with neck pain will be consecutively recruited from each clinic, for a total of 500 patients. The flow diagram can be seen in Fig. 1 and the planned schedule of enrollment, interventions, and assessments in Fig. 2.

#### Inclusion criteria, implementation evaluation

Physiotherapy clinics in primary health care with ≥3 registered physiotherapists working at the clinic.

#### Exclusion criteria, implementation evaluation

No exclusion criteria for registered physiotherapists in the clinics that are recruited in the study.

#### Inclusion criteria, effectiveness evaluation

The physiotherapists will include patients ≥18 years old with neck pain. Patients will be required to have internet access by phone, tablet, or computer, be able to read and understand Swedish, and be interested in participating in the study.

#### Exclusion criteria

Physiotherapists should exclude patients with “red flags”: symptoms that suggest a serious illness or spinal abnormality, including serious trauma to the neck and no X-ray, preceding neck surgery, osteoporosis, myelopathy, history of cancer, unexplained weight loss, current fever, history of infections, constant and progressive non-mechanical pain, insidious progression of pain, signs of spinal cord compression (neurological examination to exclude spinal cord or cervical myelopathy such as clumsy hands, altered gait, or disturbances in sexual, bladder, or sphincter function).

#### Development of the implementation strategy based on expected barriers

Barriers to implement evidence-based interventions in physiotherapy have been identified and are, for example, lack of time, lack of high-quality research, confusion, and misperceptions of the new method [19]. Identified facilitating factors are, for example, approval by management [34], outreach visits [29], and fast support and training for the users if new technology will be introduced [35]. These barriers and facilitators [19, 20, 35] have been considered in the development of the implementation activities for successful uptake of the NSEIT and NSE among physiotherapists. The barriers expected and the planned activities to overcome these are described in Table 1.

**Table 1:** Barriers and activities to minimize the barriers impact in the implementation of NSEIT

Barriers	To overcome barriers	Benefits
Physiotherapists in primary care have limited time for education and learning new methods	Before implementation starts, inform manager and acquire permission to implement NSEIT	Fewer individual visits to physiotherapists will save time
Physiotherapists need time to practice the new exercises and to master NSEIT	Inform physiotherapists and manager that some extra time is needed at the beginning	Will save time due to fewer individual visits
Patients or physiotherapists have problems with the Internet-based program (NSEIT)	The web-based platform INERA is supported by the Regions in Sweden. The physiotherapist can call for support	Fast support will facilitate the use of NSEIT
Physiotherapists forget to use NSEIT if there are long intervals between patients	Repetition of the neck-specific program after one and 3 months in group A (experimental group).	Will remind the physiotherapist to use NSEIT
Physiotherapists forget how to use neck exercises and/or NSEIT	Repetition of the neck-specific program after one and 3 months in group A (experimental group).	Repetition of instruction on how the exercise will be performed.
Physiotherapists have questions about the exercises and/or NSEIT	Support from the facilitators by chat and on-line meetings in group A (experimental group).	Enhanced confidence in using the method

### Procedure

The 20 physiotherapy clinics will be randomized to 2 groups, stratified for the number of physiotherapists working at the clinic (≥4 physiotherapists or 3 physiotherapist). A computerized block randomization list, conducted by a statistician and allocated by a project team member, will be used. The randomization will be performed by an independent researcher. The researcher sends an e-mail to all physiotherapists at the clinic, containing information about the randomization group. Due to the nature of the study can the physiotherapist not be blinded. Any change in the protocol will be communicated with the ethics committee and if approved appropriate changes will be made in the ClinicalTrial.gov Registry.

### Recruitment of physiotherapy clinics and patients

In the first step, physiotherapy clinics will be recruited through contact with health care providers and research coordinators in county councils in Sweden. In the second step, an oral on-line presentation describing the study will be given to those health care providers that express interest in the study. If the eligible physiotherapy clinic confirms participation via e-mail after the presentation, physiotherapists will be included after written informed consent. Registered physiotherapists working at the included physiotherapy clinics will screen patients for eligibility by taking a detailed medical history and careful clinical examination, following the regulations for registered physiotherapists in Sweden (Health and Medical Services Act 2017:30 [36] and the patient security law (SFS 2010: 659. Patientsäkerhetslag. Stockholm: Socialdepartementet) [37]. Patients with neck pain deemed to benefit from the NSE or the NSEIT and who are willing to participate in the study will be included. If the eligible patient confirms participation, written and informed consent will be completed through Health Care Guide 1177 (a national hub for health care information and services in Sweden) and patients logging in using BankID. Before completing the consent form, potential participants (physiotherapists and patients) will read the information, describing the purpose of the study, data collection process, duration of commitment, intervention, and benefits and harms of the treatment. The information also explains that participants can withdraw from participation at any time of the study. Patients are insured by the County Council Mutual Insurance Company, a mutual insurance company that covers patient injury (compensation to those who suffer harm) for all public health care providers and private providers contracting with the Swedish government.

#### Implementation study – education and practical training of physiotherapists

Physiotherapists in both group A (the experimental group) and group B (the control group) will receive on-line theoretical education including 3 hours of practical training by the project leaders. The standardized theoretical instruction includes three 45-minute on-line lectures (Table 2). It will be followed by 3 hours of practical training, including clinical examination in patients with neck disability and instructions on how to perform the neck-specific exercises. The project leaders developed the education program, based on the education used in our earlier studies [11, 12, 23, 38], and the physiotherapists will receive practical training from physiotherapists well used to the exercise intervention and internet-based program. The physiotherapists will be informed that NSEIT was non-inferior to NSE in the RCT and that the research findings targeted WAD grades II and III. The physiotherapist will not be prohibited from using NSEIT for other patients with neck pain. To facilitate the implementation process, group A will receive additional support: the physiotherapists will be able to contact the project leader via chat. The physiotherapists in group A will also have two on-line workshops after one and 3 months (2 × 45 minutes); see Table 2. Physiotherapists in group B will not receive additional support or education after the first three theoretical on-line lectures and the 3 hours’ practical education.

**Table 2:** Implementation component

Implementation education	Content
Lecture 1	• Introduction to the neuromuscular function of the neck
	• Current evidence for exercise in neck pain and WAD
	• Results of the previous two RCTs regarding NSEIT and NSE
	• Information about the progression of the exercise program
Lecture 2	• The whiplash injury mechanism and causes of general neck pain
	• Relevant musculoskeletal anatomy and function
	• Biopsychosocial factors in persisting pain
	• Neurophysiological and neurobiological processes underlying chronic pain
	• Strategies for dealing with neck pain relapse
	• Theoretical base for the medical history and clinical examination
	• How to measure neck muscle endurance and clinical signs of neuromuscular neck dysfunction.
Lecture 3	• Information on how to use the internet-based program
	• Practical training in the use of NSEIT in the test version
Practical training	• Face-to-face meeting with physiotherapists at the clinic
	• Practical training in clinical examination of neuromuscular dysfunction and neck muscle endurance
	• Practical training in neck-specific exercise
Workshop 1	• Discussion regarding difficulties experienced in using NSEIT/NSE and to share information with other physiotherapists; for example, regarding the progression of the exercises and patients with sustained pain.
Workshop 2	• Discussion regarding difficulties experienced in using NSEIT/NSE and to share information with other physiotherapists; for example, regarding the progression of the exercises and patients with sustained pain.

### NSE and NSEIT - the implementation object

#### Neck-specific exercise with internet support

The internet-based exercise program, NSEIT, is available on-line by phone, tablet, or computer via the Swedish National Support and Treatment Services, INERA. Since 2016, all regions in Sweden have been connected to the Support and Treatment services, a web-based e-health solution to enhance digitalization in health care. The platform is accessed through Health Care Guide 1177 and patients logging in using BankID. The physiotherapists logging in to the Support and Treatment services will choose exercises and information from two different programs, WAD ExerciseIT (for patients with whiplash-associated disorders) and Neck ExerciseIT (for patients with non-specific neck pain). The two programs contain the same neck-specific exercises and the same information about persistent pain, the benefits of exercise directed to the deep neck muscles, strategies for dealing with neck pain relapse, ergonomic advice related to the neck, complementary exercises for shoulder muscles and the low back, and strategies for continuing exercise after the treatment period. WAD ExerciseIT contains information about whiplash injury and Neck ExerciseIT has information about non-specific neck pain and stretching exercises. The physiotherapists will choose and individually progressively assign the neck-specific exercises during the treatment period. In the RCT study, each patient had 4 meetings with the physiotherapist, at weeks 1, 2, 3, and 7, to ensure that the patient performed the exercises as intended [12]. At week 1, the first visit to the physiotherapist included an introduction to the exercise intervention chosen from a clear and written framework of exercises. The physiotherapist introduced exercises aimed to facilitate activation of the deep neck muscles and information about the importance of good body and neck posture to minimize postural strain. At week 2, they introduced isometric neck-specific exercises in supine position and continued them in sitting position. Weeks 3 to 12 involved continued training with gradual progression, using a rubber band in sitting position. The patient could also exercise at a gym, using a weighted pulley or guild board. The physiotherapists will be informed about the four meetings in the RCT study, but in this implementation study the physiotherapists will be free to book appointments with the patients.

#### Neck-specific exercises with regular visits to the physiotherapy clinic

Physiotherapists will also be able to use the NSE in their clinic for patients who need or would like supervised training; all the NSE exercises and information will be available on paper. The patients will receive the same information and exercise as in the NSEIT program, but it will be delivered by a physiotherapist. Patients will not be withdrawn if they receive any other physiotherapy treatment (for example acupuncture or manual treatment) in combination with the NSEIT/NSE.

### Measurements

Outcome measurements will be recorded at baseline and after 3 and 12 months. See Fig. 2 for the planned schedule of enrollment, interventions, and assessments, and Table 3 for the relationship between the RE-AIM framework and data collection. All questionnaires will be answered electronically through a website. Physiotherapists and patients will receive a disposable code e-mailed by a project team member to log in to the System Artrologik (Survey&Reports). Patients will answer the baseline and 3-month questionnaires through the Support and Treatment services and the 12-month questionnaire through the System Artrologik. The electronic questionnaire cannot be submitted if the core outcomes are not answered. If a participant does not answer the questionnaire after two reminders (1.5 weeks after it is due and after another 1.5 weeks), a project team member not involved in the randomization will contact the participants and remind them.

**Table 3:** RE-AIM framework and data collection

Dimension	Research question	Data Collection
Reach	To what extent do patients with WAD receive NSEIT/NSE in primary care?	Primary outcome Data from the medical record from each included physiotherapy clinic and/or the web-based platform INERA
Effectiveness	Is the NSEIT/NSE program effective at decreasing disability and pain in patients with WAD or non-specific neck pain in primary care? Will the results be the same as in the RCTs at 3 and 12 months of follow-up?	Primary and secondary outcomes. Patient self-reported outcomes. The results will be compared with those of previous studies.
Adoption	To what extent do the physiotherapists apply the NSEIT/NSE to patients with WAD or non-specific neck pain?	Secondary outcomes Physiotherapist-reported outcomes. Qualitative methods
Implementation	To what extent is the intervention delivered according to the NSE protocol (fidelity)?	Qualitative methods
Maintenance	To what extent will physiotherapists be continuing to use NSEIT/NSE at the 12-month follow-up?	Secondary outcomes Physiotherapists reported outcomes Qualitative Methods

#### Implementation

Background data on each physiotherapist will be collected and include age, sex, years working as physiotherapist, and post-secondary education in physiotherapy.

#### Primary outcome

The primary outcome will be the proportion of patients with neck pain receiving neck-specific exercise during the intervention period. Data will be collected from each included physiotherapy clinic, based on each physiotherapist’s registered ICD-10 code (S134, S134A, S134B, S134C, T918, M501, M502, M503, M530, M531, M542) in the medical record.

#### Secondary outcome

The Evidence-Based Practice Attitude Scale (EBPAS) will be used to assess the physiotherapists’ attitudes to implementation of evidence-based practice [39] and will be collected only at baseline. The questionnaire includes 15 items, each answered on a 5-point scale from 0 (not at all) to 4 (agree completely). The total score ranges from 0 to 60, and higher scores indicate more favorable attitudes. The questionnaire can be divided into four different scales: scale 1 (requirements), scale 2 (appeal), scale 3 (openness), and scale 4 (divergence). Self-efficacy and confidence in diagnosing and treating patients with neck pain will be measured by use of the Practitioner Confidence Scale (PCS) [40]. The PCS will be collected before and directly after the theoretical and practical education, and at the 3- and 12-month follow-ups. A total of 4 items are reported by the practitioner and a total score is collected, where 4 represents greatest self-confidence and 20 represents lowest self-confidence.

The secondary outcome measures will also include questions about each physiotherapist’s experience of the neck-specific program: the number of times the physiotherapist had contact with the patient (visits, telephone, and/or chat) and information about additional or other exercises and/or treatment for neck pain will be collected via the medical record and/or the web-based support and treatment platform INERA, and/or through the Swedish Classification of Health Interventions [41].

#### The effectiveness of NSEIT/NSE

Background data will be collected, including age, sex, symptom duration, and whiplash injury or other reason for neck pain.

The primary outcome measure that will be collected is neck-specific function as measured by the Neck Disability Index (NDI) [42].

The secondary outcome measurements will be pain intensity (current, average last week, worst pain last week) in the neck, head, and arm, and dizziness (current, average last week, worst dizziness last week) in the past week, using a numeric rating scale (NRS, score 0 to 10: 0 = no pain or dizziness, 10 = worst imaginable pain or dizziness) [43]; answers to questions about health-related quality of life; work ability measured with the Work Ability Score (WAS) reporting “current work ability compared to highest work ability ever” (score 0 to 10; 0 = cannot work at all, 10 = best work ability [44]; and patient’s improvement or deterioration over time, measured with an 11-point global rating scale (GRS: − 5 = vastly worse, 0 = unchanged, 5 = completely recovered) [45]. Any adverse events or negative effects will be registered at the 3-month follow-up.

#### Qualitative study

To further evaluate the implementation strategy, two qualitative studies will be conducted. Data will be collected from semi-structured focus group discussions with a purposeful sample of physiotherapists who have participated in the study. Physiotherapists from both groups—group A with increased implementation support, and group B with no further support after the first three on-line instructional sessions and one practical instructional session—will be included. Physiotherapists of both sexes and varied ages and professional experiences will be sought. Six focus group discussions with 3–5 physiotherapists in each group will be held 1–2 months after the implementation activity and approximately 1 year after. The focus group discussions will be conducted digitally as a video conference meeting with a secure digital system (Visiba care). Data will be analyzed with qualitative content analysis and an inductive approach for category development, and alternatively with a deductive-inductive approach [46].

The interview guides will include questions about, for example, the physiotherapist’s experiences instructing patients in neck-specific training, which patients they have treated using the neck-specific exercises programs, experiences dealing with patients with neck pain after the implementation of neck-specific exercises, and experiences with implementation support.

### Statistical analyses

All analyses will be conducted in collaboration with a statistician. Data will be analyzed according to intention to treat and supplemented with per protocol analysis. An analysis will be done of the missing data. Imputation methods may be used when deemed to have additional value. Subgroup analyses of age, gender, neck pain intensity, headache, and dizziness may possibly be performed.

- *Data regarding the implementation strategy* will be analyzed using a parametric t-test and a linear mixed model or repeated-measures ANOVA or the non-parametric Mann-Whitney U test and Kruskal-Wallis test, depending on the data. Analyses will evaluate differences between groups A and B and changes in variables over time.
- *Data regarding the effectiveness of NSEIT and NSE* will be analyzed with a linear mixed model or repeated-measures ANOVA or Kruskal-Wallis, depending on the data. NDI, pain intensity, headache, and dizziness will be evaluated as dependent variables with time as an independent fixed factor (baseline, 3 months, and 12 months after start of intervention). The results will be compared with the results from the RCT studies [8, 12].

The effect of neck-specific exercises in the present study for WAD grades II and III and for patients with other neck-pain disorders will also be evaluated and compared with the results in the RCT studies [8, 12].

### Sample size

Sample size and power regarding group differences were calculated by a statistician. The aim of the study is to evaluate both implementation and effectiveness. The sample size calculation to evaluate implementation was based on the assumption that 15% more patients will receive neck-specific exercises in the intervention group (40% in the intervention group and 25% in the control group). The required sample size under individual randomization will be 150 patients in each arm. With 10 physiotherapy clinics (clusters) in each arm (a total of 20 clusters), intra-cluster correlation of 0.02 and a cluster size of 21 patients, a total of 420 patients will be recruited for 80% power and a significance level of 0.05. To ensure that enough people are in each group after dropouts, a total of 500 patients will be included, 25 patients from each cluster.

Sample size calculation to evaluate effectiveness was based on a clinically relevant improvement of 7% in the NDI, an effect size of 0.2 (Cohen’s f) or 0.4 (Cohen’s d), a correlation among repeated measures of 0.3, and the need for a total of 56 participants for 80% power and a significance level of 0.05. With an expected dropout rate of 20%, a total of 70 participating patients will be needed. The total number of patients may be more than 70 because patients will be recruited at each clinic.

Repeated-measure ANOVA or a linear mixed model will be used to examine changes in the patient-reported outcomes over time (i.e., at baseline and 3 and 12 months). The results will be compared with the results from the RCT studies [8, 12]. Patients who are included in both the intervention and control implementation groups and receive neck-specific exercises will be treated as one group in terms of the effect of the intervention. Analyses will be performed with parametric statistics if assumptions of normality are met, and otherwise with non-parametric statistics.

### Data management

All data will be monitored by the project team, independent on the study sponsor. To ensure participant confidentiality will a unique identifier be assigned to each participant after enrolment. Data will be stored on a secure website in the County Council of Sörmland. The website is password protected and will only be accessible by the project team. The results will be presented at the group level, and no connection to the individual person can be made. Outcome assessors and data analysts will be blinded for intervention allocation by having a researcher not involved in the analyses recode the groups using two random numbers.

### Dissemination policy

The results of the study will be disseminated through scientific papers, seminars and popular science reports and stakeholder. Authorship guidelines include actively contributing to scientific papers and professional writers will not be used.

### Timeline

**Fig. 3:**
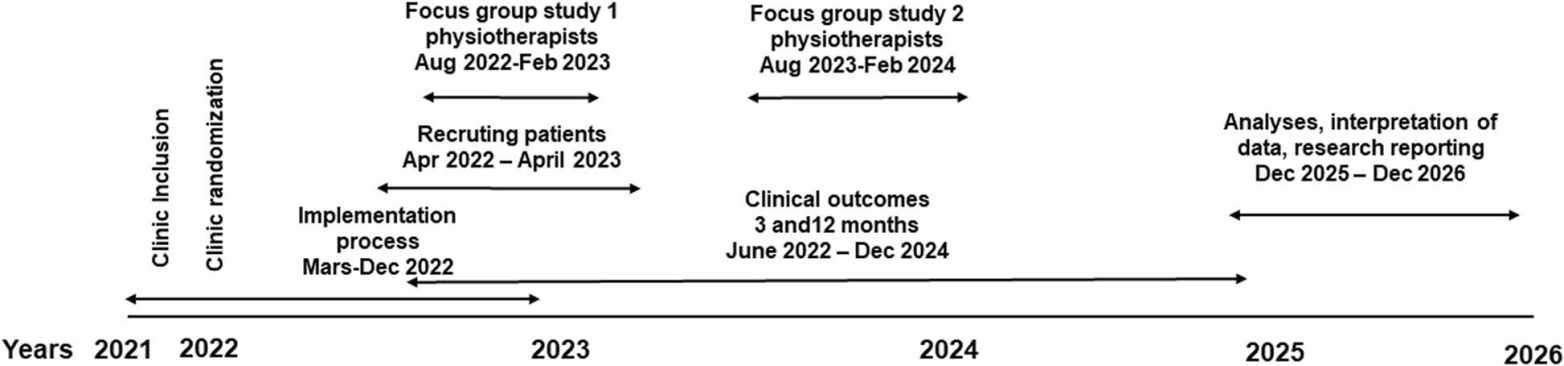
Timetable for recruitment, implementation, and outcomes

Planning of the study began in January 2021. The trial started in April, 05, 2022 and we estimate that patients will be enrolled in the study in 2022–2023, with follow-up continuing until December 2024 (Fig. 3).

## Discussion

Neck-specific exercises have been evaluated in two RCTs, showing reduced disability and neck pain in individuals with persistent WAD [8, 9, 12]. Access to internet-based treatment can shorten waiting time, enhance availability, and reduce costs. NSEIT was noninferior to NSE, and the exercise program can be delivered via internet with fewer visits to health care. The next step will be to implement NSEIT among physiotherapists in primary care. This pragmatic project is based on the RE-AIM framework and diffusion-of-innovation theory. At the staff and setting levels, our intent to evaluate the approach’s reach (the proportions of patients receiving NSEIT/NSE), and the adoption, implementation, and maintenance of the method. At the patient level, we plan to evaluate the effectiveness of NSEIT/NSE for patients with persistent WAD, the diffusion of NSEIT/NSE to other patients with neck pain, and the maintenance of results (long-term effectiveness). The project is important because it will increase our understanding of the factors that will promote implementation of NSEIT/NSE in primary care and because it will evaluate the effectiveness of the method. Results from this study will bridge the gap between research and clinical practice and guide large-scale implementation of NSEIT/NSE.

### Limitations

The limitations of the present study are related to the pragmatic approach. To study the implementation process and the adoption, diffusion, and maintenance of the NSEIT/NSE in clinical practice we could not follow as strict a protocol as in an RCT, which means we have less control over the study. The physiotherapists will select patients whose condition they have deemed to be improved by neck-specific exercises. Because all patients will be evaluated as one group, with no comparison with other treatments or exercise methods, threats to internal validity exist; that is, threats to confidence that the results are trustworthy. However, we will compare the results with the findings in our previous RCTs. Threats to the study’s external validity arise from the fact that only neck-pain patients and physiotherapy clinics that are interested in participating in the study will be evaluated. With these concerns in mind, the results can be generalized to physiotherapy clinics in primary care, as many different regions and physiotherapy clinics will participate in the study.

## Data Availability

Not applicable; the study corresponding to this manuscript is in the recruitment phase.

## Abbreviations

WAD: Whiplash-associated disorders
RCT: Randomized controlled trial
NSEIT: Neck-specific exercise with internet support
NSE: Neck-specific exercises at a physiotherapist clinic 2 times a week over 12 weeks
NDI: Neck Disability Index
EBM: Evidence-based methods
WAD ExerciseIT: The web-based information and exercise program directed at patients with WAD
Neck ExerciseIT: The web-based information and exercise program directed at patients with non-specific neck pain
ICD-10: The 10th revision of the International Statistical Classification of Diseases and Related Health Problems
EBPAS: Evidence-Based Practice Attitude Scale
PCS: Practitioner Confidence Scale
INERA: The Swedish web-based National Support and Treatment Services
NRS: Numeric rating scale
WAS: Work Ability Score
GRS: Global rating scale
ANOVA: Analysis of variance

## References

[1] Hanney SR, Castle-Clarke S, Grant J, Guthrie S, Henshall C, Mestre-Ferrandiz J (2015). How long does biomedical research take? Studying the time taken between biomedical and health research and its translation into products, policy, and practice. Health Res Policy Syst.

[2] Carroll LJ, Holm LW, Hogg-Johnson S, Cote P, Cassidy JD, Haldeman S (2009). Course and prognostic factors for neck pain in whiplash-associated disorders (WAD): results of the bone and joint decade 2000-2010 task force on neck pain and its associated disorders. J Manip Physiol Ther.

[3] Sterling M (2014). Physiotherapy management of whiplash-associated disorders (WAD). Aust J Phys.

[4] Carragee EJ, Hurwitz EL, Cheng I, Carroll LJ, Nordin M, Guzman J (2008). Treatment of neck pain: injections and surgical interventions: results of the bone and joint decade 2000-2010 task force on neck pain and its associated disorders. Spine (Phila Pa 1976).

[5] Rebbeck T (2017). The role of exercise and patient education in the noninvasive Management of Whiplash. J Orthop Sports Phys Ther.

[6] Michaleff ZA, Maher CG, Lin CW, Rebbeck T, Jull G, Latimer J (2014). Comprehensive physiotherapy exercise programme or advice for chronic whiplash (PROMISE): a pragmatic randomised controlled trial. Lancet.

[7] Stewart MJ, Maher CG, Refshauge KM, Herbert RD, Bogduk N, Nicholas M (2007). Randomized controlled trial of exercise for chronic whiplash-associated disorders. Pain.

[8] Ludvigsson ML, Peterson G, Dedering A, Peolsson A (2016). One- and two-year follow-up of a randomized trial of neck-specific exercise with or without a behavioural approach compared with prescription of physical activity in chronic whiplash disorder. J Rehabil Med.

[9] Peterson G, Nilsson D, Trygg J, Peolsson A (2018). Neck-specific exercise improves impaired interactions between ventral neck muscles in chronic whiplash: a randomized controlled ultrasound study. Sci Rep.

[10] Ludvigsson ML, Peterson G, O'Leary S, Dedering A, Peolsson A (2015). The effect of neck-specific exercise with, or without a behavioral approach, on pain, disability, and self-efficacy in chronic whiplash-associated disorders: a randomized clinical trial. Clin J Pain.

[11] Peolsson A, Landen Ludvigsson M, Tigerfors AM, Peterson G (2016). Effects of neck-specific exercises compared to waiting list for individuals with chronic whiplash-associated disorders: a prospective, randomized controlled study. Arch Phys Med Rehabil.

[12] Peolsson A, Landen Ludvigsson M, Peterson G (2017). Neck-specific exercises with internet-based support compared to neck-specific exercises at a physiotherapy clinic for chronic whiplash-associated disorders: study protocol of a randomized controlled multicentre trial. BMC Musculoskelet Disord.

[13] Suman A, Dikkers MF, Schaafsma FG, van Tulder MW, Anema JR (2016). Effectiveness of multifaceted implementation strategies for the implementation of back and neck pain guidelines in health care: a systematic review. Implement Sci.

[14] Lokker C, McKibbon KA, Colquhoun H, Hempel S (2015). A scoping review of classification schemes of interventions to promote and integrate evidence into practice in healthcare. Implement Sci.

[15] Rebbeck T, Maher CG, Refshauge KM (2006). Evaluating two implementation strategies for whiplash guidelines in physiotherapy: a cluster randomised trial. Aust J Physiother.

[16] Fritz J, Wallin L, Soderlund A, Almqvist L, Sandborgh M (2020). Implementation of a behavioral medicine approach in physiotherapy: impact and sustainability. Disabil Rehabil.

[17] Leeman J, Birken SA, Powell BJ, Rohweder C, Shea CM (2017). Beyond "implementation strategies": classifying the full range of strategies used in implementation science and practice. Implement Sci.

[18] Macdermid JC, Miller J, Gross AR (2013). Knowledge translation tools are emerging to move neck pain research into practice. Open Orthop J.

[19] Scurlock-Evans L, Upton P, Upton D (2014). Evidence-based practice in physiotherapy: a systematic review of barriers, enablers and interventions. Physiotherapy.

[20] Golden SD, Earp JA (2012). Social ecological approaches to individuals and their contexts: twenty years of health education & behavior health promotion interventions. Health Educ Behav.

[21] Valente TW, Rogers EM (1995). The origins and development of the diffusion of innovations paradigm as an example of scientific growth. Sci Commun.

[22] Glanz K, Rimer BK, Lewis FM (2002). Health behavior and health education: theory, research, and practice.

[23] Carlfjord S, Landen Ludvigsson M, Peolsson A, Peterson G. Adoption of a research-based program for neck disorders implemented in primary care physiotherapy: a short- and long-term follow-up survey study. Physiother Theory Pract. 2021;37(1):89-98. 10.1080/09593985.2019.1608610 31030585

[24] Forsetlund L, Bjorndal A, Rashidian A, Jamtvedt G, O'Brien MA, Wolf F (2009). Continuing education meetings and workshops: effects on professional practice and health care outcomes. Cochrane Database Syst Rev.

[25] Ritchie C, Smith A, Sterling M (2020). Medical and allied health service use during acute and chronic post-injury periods in whiplash injured individuals. BMC Health Serv Res.

[26] Vikne J, Oedegaard A, Laerum E, Ihlebaek C, Kirkesola G (2007). A randomized study of new sling exercise treatment vs traditional physiotherapy for patients with chronic whiplash-associated disorders with unsettled compensation claims. J Rehabil Med.

[27] Sterling M, Vicenzino B, Souvlis T, Connelly LB (2015). Dry-needling and exercise for chronic whiplash-associated disorders: a randomized single-blind placebo-controlled trial. Pain.

[28] Landen Ludvigsson M, Peolsson A, Peterson G, Dedering A, Johansson G, Bernfort L (2017). Cost-effectiveness of neck-specific exercise with or without a behavioral approach versus physical activity prescription in the treatment of chronic whiplash-associated disorders: analyses of a randomized clinical trial. Medicine (Baltimore).

[29] Fritz J, Wallin L, Soderlund A, Almqvist L, Sandborgh M (2019). Implementation of a behavioral medicine approach in physiotherapy: a process evaluation of facilitation methods. Implement Sci.

[30] Glasgow RE, Vogt TM, Boles SM (1999). Evaluating the public health impact of health promotion interventions: the RE-AIM framework. Am J Public Health.

[31] Glasgow RE, Harden SM, Gaglio B, Rabin B, Smith ML, Porter GC (2019). RE-AIM planning and evaluation framework: adapting to new science and practice with a 20-year review. Front Public Health.

[32] Pinnock H, Barwick M, Carpenter CR, Eldridge S, Grandes G, Griffiths CJ (2017). Standards for reporting implementation studies (StaRI): explanation and elaboration document. BMJ Open.

[33] Curran GM, Bauer M, Mittman B, Pyne JM, Stetler C (2012). Effectiveness-implementation hybrid designs: combining elements of clinical effectiveness and implementation research to enhance public health impact. Med Care.

[34] Driessen MT, Groenewoud K, Proper KI, Anema JR, Bongers PM, van der Beek AJ (2010). What are possible barriers and facilitators to implementation of a participatory ergonomics programme?. Implement Sci.

[35] Cresswell KM, Bates DW, Sheikh A (2013). Ten key considerations for the successful implementation and adoption of large-scale health information technology. J Am Med Inform Assoc.

[36] Health and Medical Services Act 2017:30. https://www.riksdagen.se/sv/dokument-lagar/dokument/svensk-forfattningssamling/halso%2D%2Doch-sjukvardslag-201730_sfs-2017-30 Retrieved 21 Feb, 2022.

[37] Patient security law, Sweden. https://www.riksdagen.se/sv/dokument-lagar/dokument/svensk-forfattningssamling/patientsakerhetslag-2010659_sfs-2010-659 Retrieved 20 Aug 2021.

[38] Peolsson A, Landen Ludvigsson M, Overmeer T, Dedering A, Bernfort L, Johansson G (2013). Effects of neck-specific exercise with or without a behavioural approach in addition to prescribed physical activity for individuals with chronic whiplash-associated disorders: a prospective randomised study. BMC Musculoskelet Disord.

[39] Skavberg Roaldsen K, Halvarsson A (2019). Reliability of the Swedish version of the evidence-based practice attitude scale assessing physiotherapist's attitudes to implementation of evidence-based practice. PLoS One.

[40] Smucker DR, Konrad TR, Curtis P, Carey TS (1998). Practitioner self-confidence and patient outcomes in acute low back pain. Arch Fam Med.

[41] The National Board of Health and Welfare Klassifikation av vårdåtgärder (KVÅ) - Socialstyrelsen Retrieved 1 Aug 2021.

[42] Vernon H (2008). The neck disability index: state-of-the-art, 1991-2008. J Manip Physiol Ther.

[43] Cleland JA, Childs JD, Whitman JM (2008). Psychometric properties of the neck disability index and numeric pain rating scale in patients with mechanical neck pain. Arch Phys Med Rehabil.

[44] El Fassi M, Bocquet V, Majery N, Lair ML, Couffignal S, Mairiaux P (2013). Work ability assessment in a worker population: comparison and determinants of work ability index and work ability score. BMC Public Health.

[45] Kamper SJ, Maher CG, Mackay G (2009). Global rating of change scales: a review of strengths and weaknesses and considerations for design. J Man Manip Ther.

[46] Hsieh HF, Shannon SE (2005). Three approaches to qualitative content analysis. Qual Health Res.

